# Supplementary material for: Tropical-leaning Atlantic Oscillation favors more typhoons toward Asian high-latitude cities
Source: Nat Commun. 2025 Dec 31;17:1177. doi: 10.1038/s41467-025-67946-4 (PMC12858986; doi:10.1038/s41467-025-67946-4)
Supplement: Supplementary file 1 — Supplementary Information [file 41467_2025_67946_MOESM1_ESM.pdf]

Supporting Information for

Tropical-leaning Atlantic Oscillation favors more typhoons toward Asian high-latitude cities

* Corresponding author: hucd@zju.edu.cn

This PDF file includes:

- Supplementary Methods
- Supplementary Tables 1 to 4
- Supplementary Figs. 1 to 26
- Supplementary References

Supplementary Methods

Data

Monthly mean precipitation data with a resolution of 2.5° × 2.5° were mainly obtained from the Global Precipitation Climatology Project (GPCP) version 2.3[1], available at https://www.ncei.noaa.gov/data/global-precipitation-climatology-project-gpcp-monthly/. Monthly mean outgoing longwave radiation (OLR) data with a resolution of 1° × 1° were mainly obtained from the NOAA's Outgoing Longwave Radiation - Daily Climate Data Record (OLR - Daily CDR): PSL Interpolated Version[2], available at https://psl.noaa.gov/mddb2/showDataset.html?datasetID=37. The AMO index is from https://climatedataguide.ucar.edu/sites/default/files/2022-03/amo_monthly.txt. The PDO index is from https://www.ncei.noaa.gov/pub/data/cmb/ersst/v5/index/ersst.v5.pdo.dat. The Indian Ocean Dipole (IOD) index is from https://psl.noaa.gov/data/timeseries/month/DMI/.

MT and TUTT longitudes

The TUTT longitude is defined as the western boundary of the zero contour of 200-hPa zonal wind speed over 5°N–20°N[3]. The MT longitude is defined as the eastern boundary of 3×10^{-6} s^{-1} at 850-hPa over 0°–20°N[4].

Bootstrap test

To estimate the uncertainty of the regression coefficients and correlation for multiple linear regression, a nonparametric bootstrap resampling method[5] was applied to the data during 1979–2023 (all years included). For each of 5000 bootstrap iterations, annual samples (corresponding to 45 years) were randomly resampled with replacement from the original dataset to form a pseudo-sample of equal size. Data are allowed to be selected repeatedly. Multiple linear regression was then performed on each resampled dataset to obtain a new set of regression coefficients and the corresponding correlation between the predicted and observed values. The distribution of these 5000 estimates was used to compute the 95% confidence intervals, defined by the 2.5th and 97.5th percentiles.

Supplementary Table 1. Observed relationship between TTD and TAO/NAO from 1979 to 2023. We use ERA5 reanalysis datasets and three tropical cyclone best-track datasets (see Methods). We perform two group of analysis, with and without three extreme El Niño years (1982, 1997 and 2015), respectively. We calculated the relationship between TAO and TTD with maximum covariance analysis (MCA), and regions are the same as that in EOF analysis. Patterns that excluding El Niño years are shown in Supplementary Fig. 7. Correlation coefficients reaching the 99% confidence level of Student's *t*-test are marked with "*".

R	TTD (Excluded El Niño years)			TTD (Included El Niño years)		
	JTWC	CMA	JMA	JTWC	CMA	JMA
TAO (MCA)	**0.73***	**0.66***	**0.64***	**0.70***	**0.66***	**0.64***
NAO	0.20	0.14	0.17	0.18	0.17	0.14

Supplementary Table 2. Relationship between preceding winter/spring SST modes and TAO/TTD. Correlation coefficients of preceding winter (December–January–February, DJF) and spring (March–April–May, MAM) Niño3.4, AMO, PDO, IOD with spring TAO and peak season (July–August–September, JAS) TTD during 1979–2023. None of these correlations is significant at the 90% confidence level of Student's *t*-test. Here the definitions of AMO, PDO and IOD indices are shown in Supplementary Methods.

Season of SST indices	R	Niño3.4	AMO	PDO	IOD
DJF	TAO_MAM	−0.19	0.15	0.01	−0.16
	TTD_JAS	−0.12	0.03	0.00	−0.05
MAM	TAO_MAM	−0.12	−0.18	0.19	−0.18
	TTD_JAS	−0.04	−0.13	0.11	−0.08

Supplementary Table 3. Definition of indices used in this manuscript. In this study, we use various indices during 1979–2023, including the TAO, NAO, NAT, PMM, TNA, PASO, NAJS, Northern Annular Mode (NAM), North Pacific Oscillation (NPO), and Arctic sea-ice concentration (SIC). Seasons are abbreviated by the first letter of the month. All the subscripts denote the regionally or zonally averaged and standardized variables.

Index	Variable (Season)	Definition
TAO	Sea level pressure (MAM)	$SLP_{(25°W–105°W, 15°N–35°N)} - SLP_{(45°W–95°W, 55°N–65°N)}$
NAO	Sea level pressure (MAM)	$SLP_{(60°W–30°E, 40°N)} - SLP_{(60°W–30°E, 70°N)}$
NAT	Sea surface temperature (AMJ)	$SST_{(30°W–50°W, 45°–55°N)} - SST_{(40°W–100°W, 25°N–40°N)} - SST_{(10°W–60°W, 7.5°N–22.5°N)}$
PMM	Sea surface temperature (JAS)	$SST_{(160°E–175°W, 8°N–18°N)} + SST_{(110°W–130°W, 14°N–20°N)}$
TNA	Sea surface temperature (JAS)	$SST_{(80°W–20°E, 0°–20°N)}$
PASO	Sea level pressure (JAS)	First leading principal component of $SLP_{(110°E–30°W, 10°N–30°N)}$
NAJS	200-hPa zonal wind (MAM)	$U200_{(40°W–105°W, 42.5°N–52.5°N)} - U200_{(40°W–105°W, 22.5°N–32.5°N)}$
NAM	Sea level pressure (MAM)	$SLP_{(0°–360°, 45°N)} - SLP_{(0°–360°, 80°N)}$
NPO	Sea level pressure (MAM)	$SLP_{(160°E–140°W, 30°N)} - SLP_{(170°E–120°W, 65°N)}$
SIC	Sea ice concentration (FMA)	$SIC_{(160°W–180°, 60°N–68°N)}$

Supplementary Table 4. List of CMIP6 models used in the analysis. The first column is the number of model. The second column shows the model's name. The third column shows the institution and the country.

Number	Name	Institution
1	ACCESS-ESM1-5	Commonwealth Scientific and Industrial Research Organization (CSIRO), Australia
2	BCC-CSM2-MR	Beijing Climate Center, China
3	CanESM5-CanOE	Canadian Centre for Climate Modelling and Analysis, Environment and Climate Change Canada, Canada
4	CAS-ESM2-0	Chinese Academy of Sciences, China
5	CESM2-WACCM	National Center for Atmospheric Research, USA
6	CIESM	Tsinghua University, China
7	EC-Earth3-Veg	Consortium of various institutions from Spain, Italy, Denmark, Finland, Germany, Ireland, Portugal, Netherlands, Norway, the United Kingdom, Belgium, and Sweden
8	FGOALS-f3-L	Chinese Academy of Sciences, China
9	FGOALS-g3	
10	FIO-ESM-2-0	First Institute of Oceanography, Ministry of Natural Resources, China, Qingdao National Laboratory for Marine Science and Technology, China
11	GFDL-CM4	National Oceanic and Atmospheric Administration, Geophysical Fluid Dynamics Laboratory, USA
12	GISS-E2-1-H	National Aeronautics and Space Administration Goddard Institute for Space Studies, USA
13	INM-CM4-8	Institute for Numerical Mathematics, Russian Academy of Science, Russia
14	INM-CM5-0	
15	IPSL-CM6A-LR	Institut Pierre Simon Laplace, France
16	KACE-1-0-G	National Institute of Meteorological Sciences/Korea Meteorological Administration, Korea
17	KIOST-ESM	Korea Institute of Ocean Science and Technology, Korea
18	MCM-UA-1-0	University of Arizona, USA
19	MIROC6	Consortium of various institutions from Japan
20	MIROC-ES2L	
21	MPI-ESM1-2-HR	Max Planck Institute for Meteorology, Germany
22	MRI-ESM2-0	Meteorological Research Institute, Japan
23	NESM3	Nanjing University of Information Science and Technology, China
24	NorESM2-LM	Norwegian Meteorological Institute, Norway
25	TaiESM1	Research Center for Environmental Changes, Taiwan
26	UKESM1-0-LL	The Met Office Hadley Centre, UK

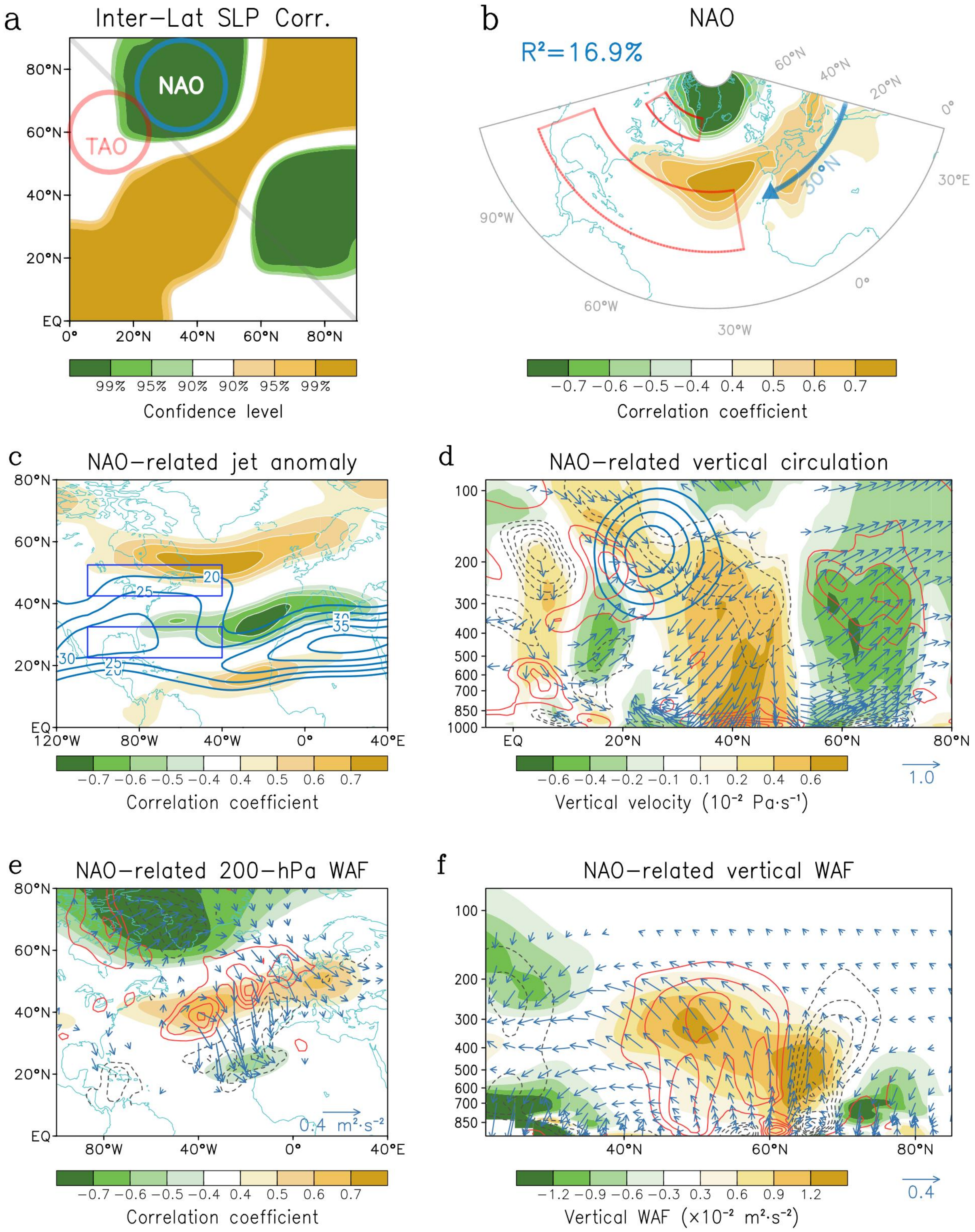

Supplementary Fig. 1. NAO dynamics. Same as Fig. 1 but for NAO, **a** is averaged between 60°W–30°E, and vertical section in **d**, **f** is averaged between 50°W–30°E.

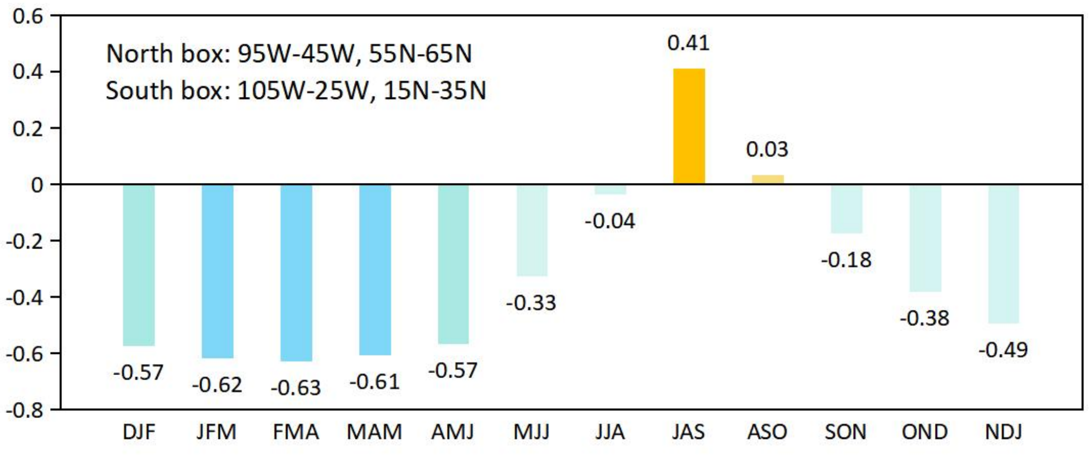

Supplementary Fig. 2. Seasonality of TAO mode. Correlation coefficient between North and South boxes of TAO in 3-month-averaged seasons during 1979–2023. The 95% confidence level threshold is 0.31.

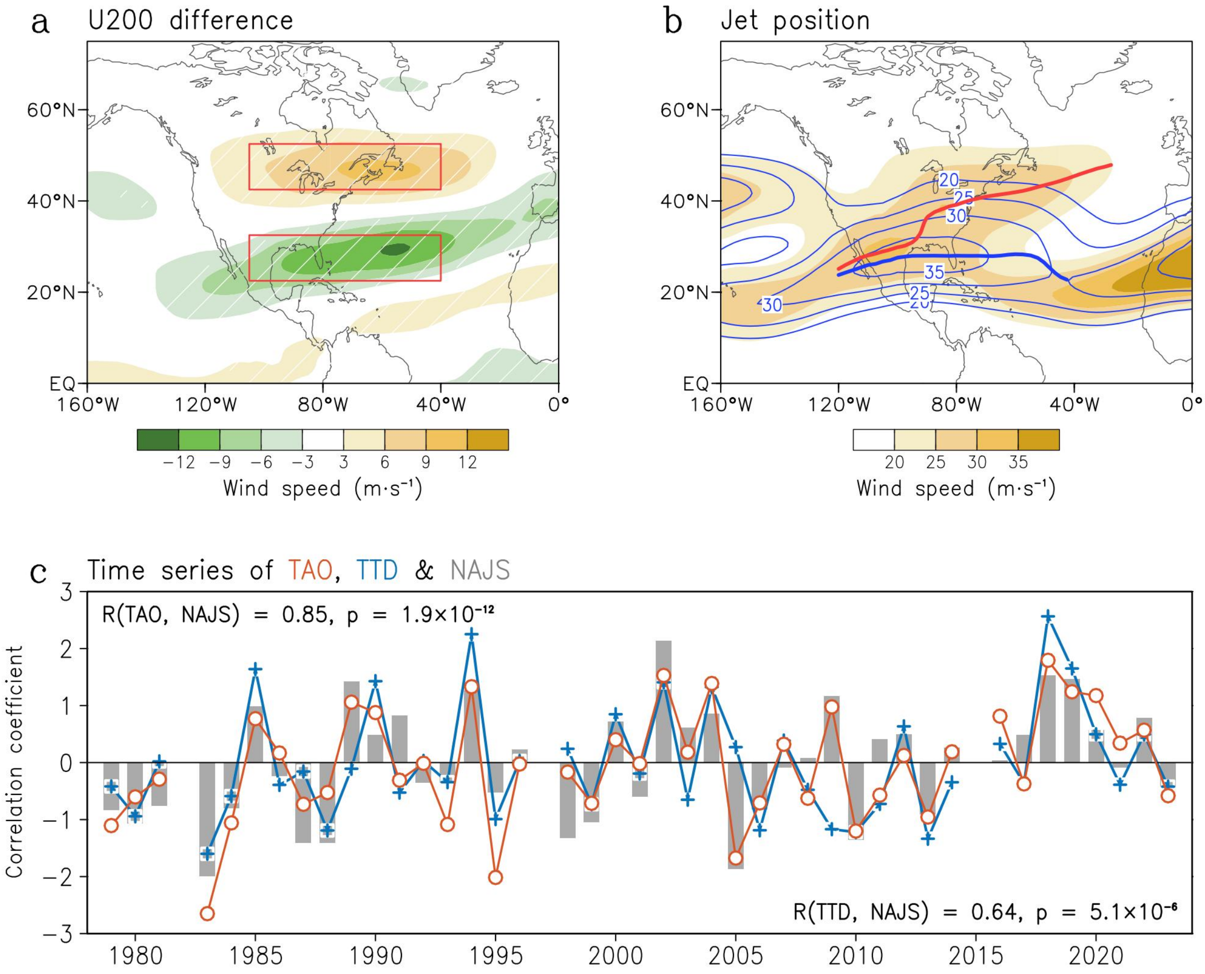

Supplementary Fig. 3. Definition of the NAJS index. a Difference in 200-hPa zonal wind between positive and negative phases of the jet meridional shift index, defined based on one standard deviation. Positive years are 1979, 1980, 1981, 1983, 1984, 1987, 1988, 1998, 1999, 2005, 2006, 2010, and 2013. Negative years are 1985, 1989, 1991, 1994, 2000, 2002, 2004, 2009, 2018, 2019, and 2022. **b** Composite of 200-hPa zonal wind speed during positive years (contours and thick blue line) and negative years (shading and thick red line). The thick lines indicate the jet axis, defined as the zero line of the meridional gradient of zonal wind. **c** Time series of TAO (red), TTD (blue) and NAJS (gray) and correlation coefficients between them.

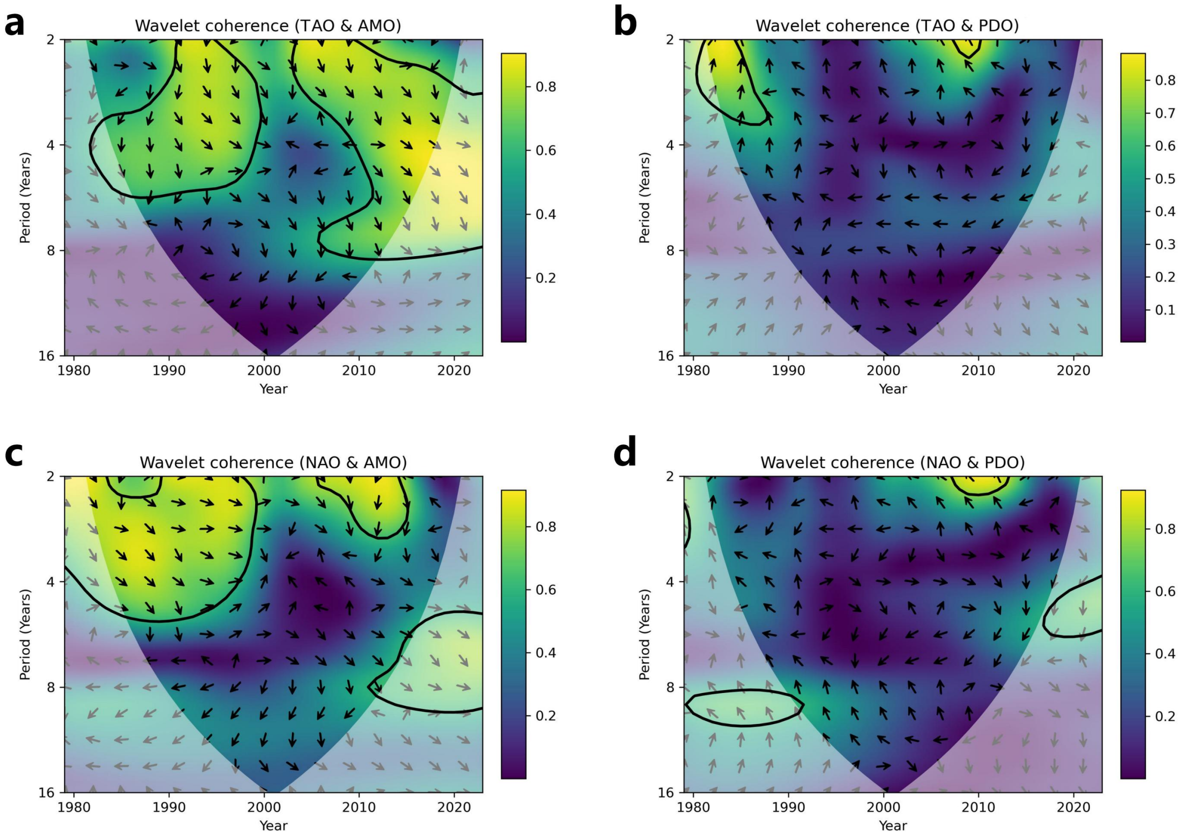

Supplementary Fig. 4. Wavelet coherence analysis of multiple modes. Wavelet coherence analysis **a** between TAO index and AMO index, **b** between TAO index and PDO index, **c** between NAO index and AMO index, and **d** between NAO index and PDO index, respectively. Contours denote passing the 95% confidence level. Vectors denote the phase relationship of two indices.

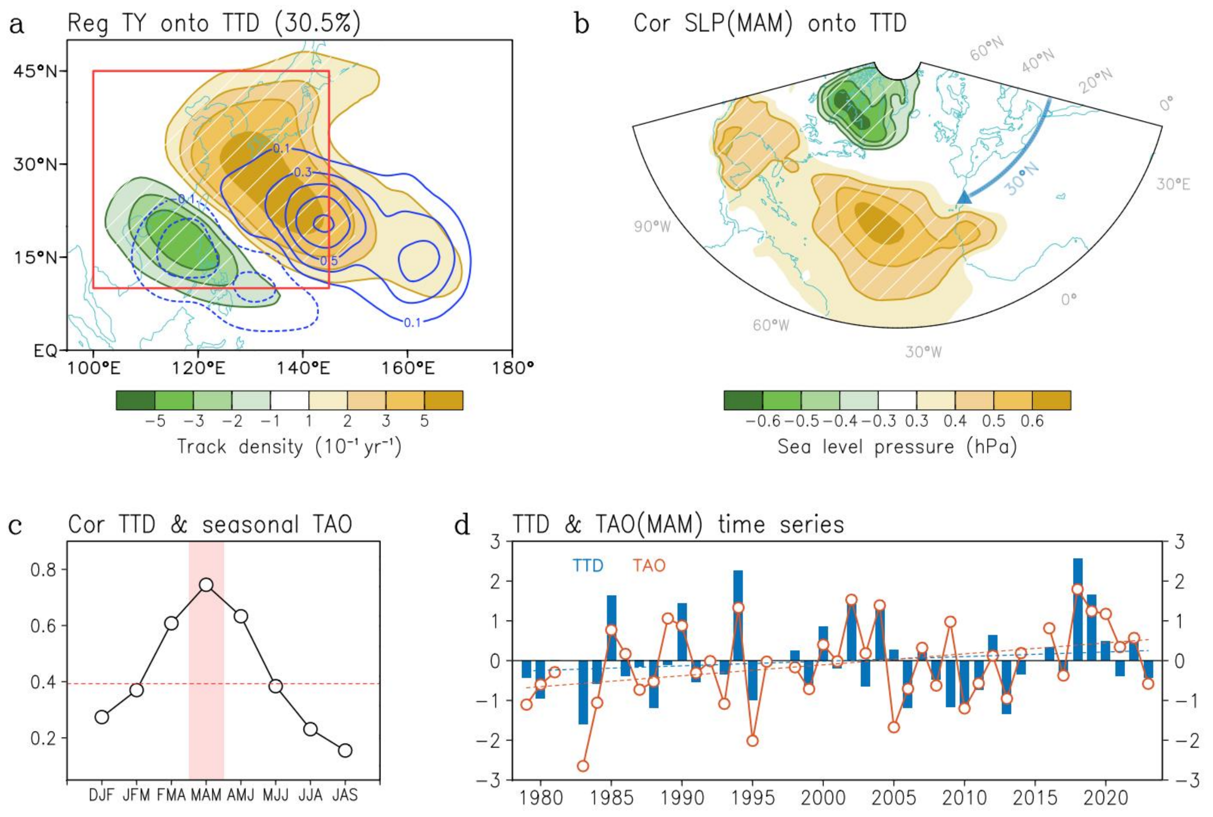

Supplementary Fig. 5. Relationship between dipolar mode of East Asian typhoon and TAO. a Regressions of peak season typhoon genesis (contour) and track density (shading) onto the TTD index during 1979–2023. Inside the parenthesis is the explained variance of the first EOF mode. **b** Regression (shading) and correlation (contour) of spring sea level pressure onto the TTD. Hatching indicates the region passing the 99% confidence level. Boxes are the regions used to define the TAO. **c** Lead-lag correlation of seasonal TAO onto the TTD. The horizontal dashed line denotes the threshold of the 99% confidence level. **d** Time series of the TTD (blue) and TAO (red) with dashed lines showing the linear trends.

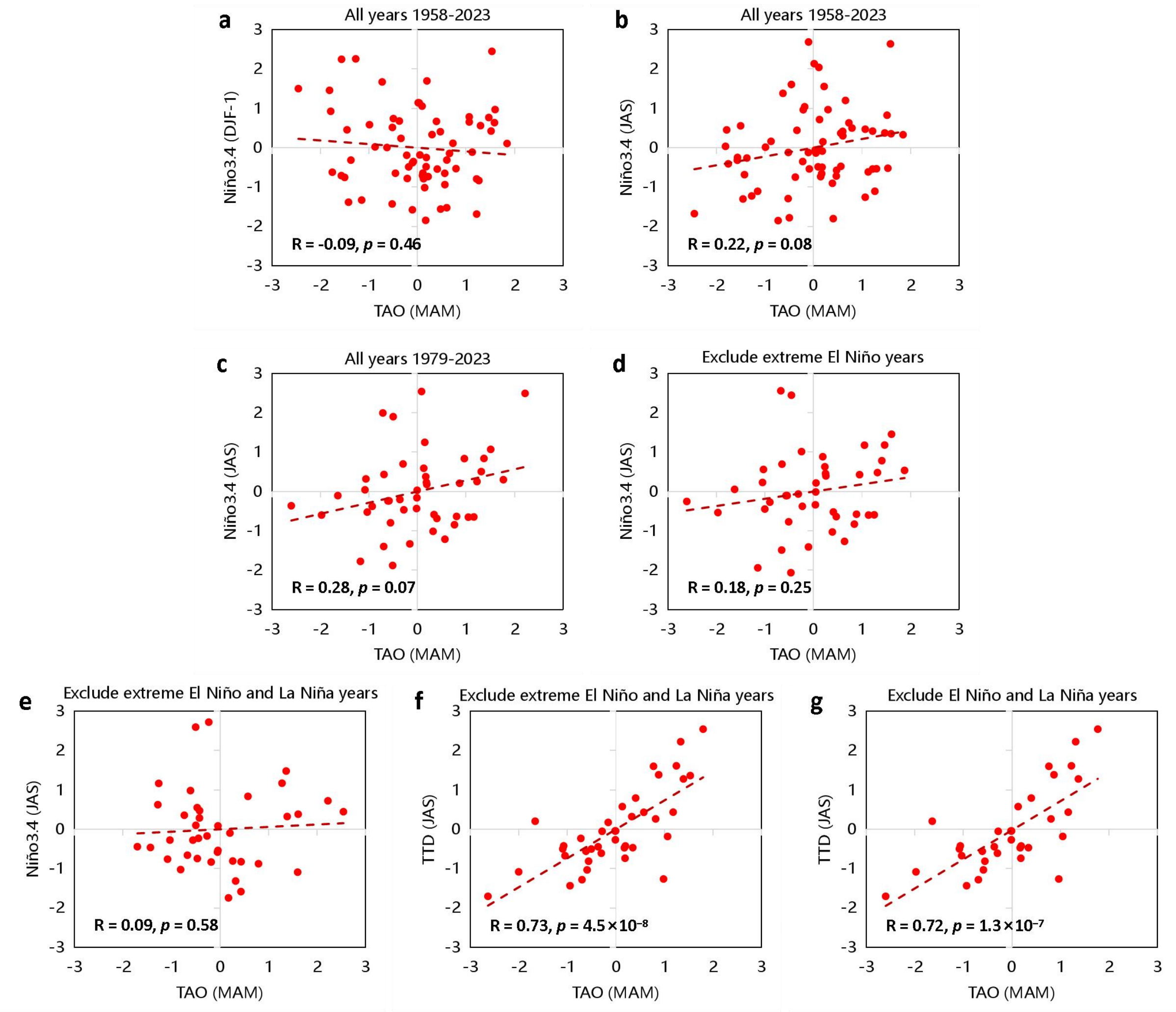

Supplementary Fig. 6. Relationship between ENSO and TAO. a Scatter plot of spring TAO versus preceding winter Niño3.4 index during 1958–2023. **b** Same as **a** but for peak season Niño3.4 index. **c** Same as **b** but during 1979–2023. **d** Same as **c** but excluding years 1982, 1997 and 2015. **e** Same as **d** but further excluding extreme La Niña years of 1988, 1999 and 2010 (selected by three lowest Niño3.4 index years). **f** Same as **e** but for TAO versus TTD. **g** Scatter plot of spring TAO versus TTD excluding El Niño and La Niña years that Niño3.4 index exceeding 1.0 absolute value. Correlation coefficients and their p-values are marked.

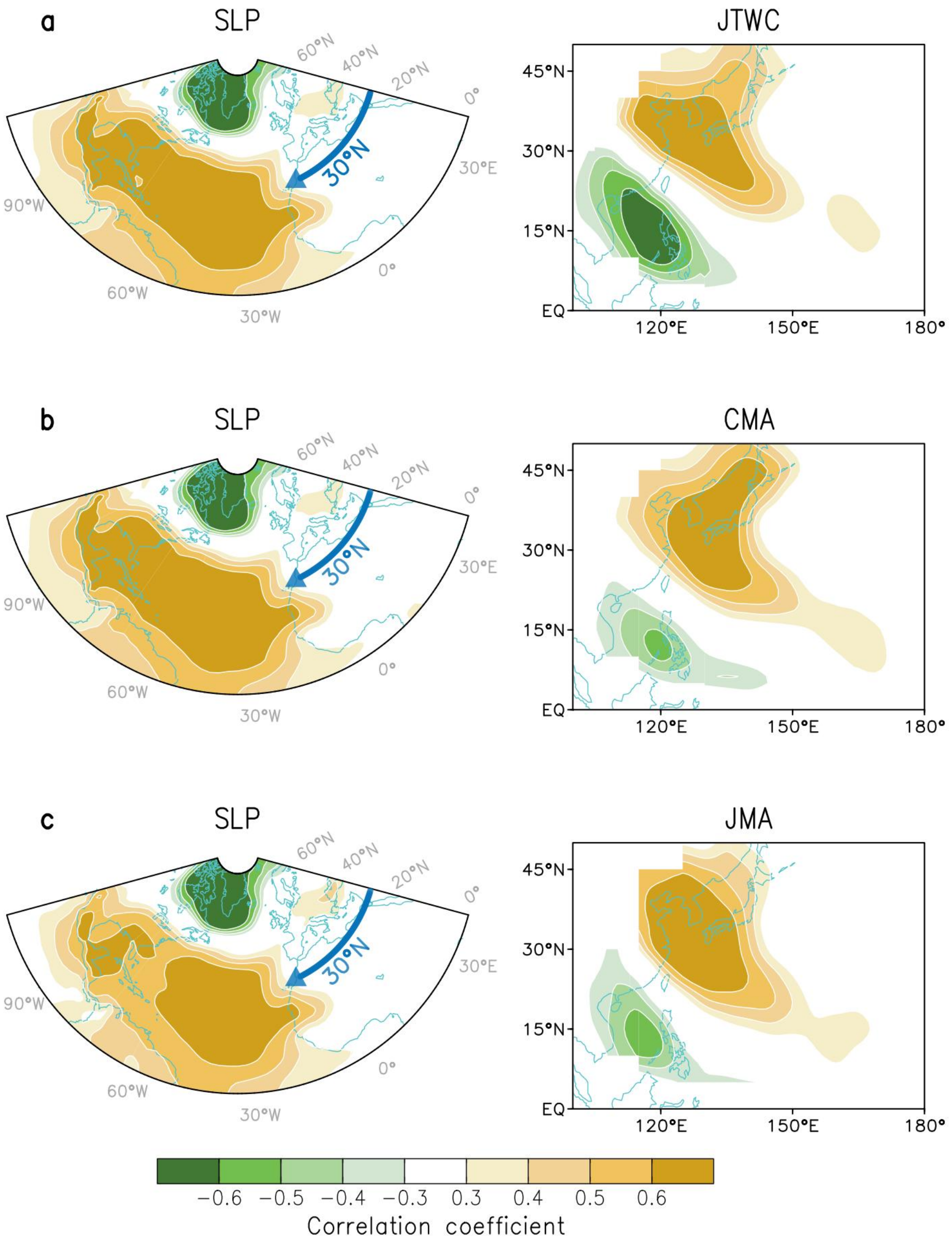

Supplementary Fig. 7. Confirmation of TAO-TTD relationship. Maximum covariance analysis (MCA) for SLP (105°W–25°W, 10°N–80°N) from ERA5 datasets and typhoon track density (100°E–145°E, 10°N–45°N) from JTWC (**a**), CMA (**b**) and JMA (**c**) tropical cyclone best-track datasets during 1979–2023. Data are normalized and exclude three El Niño years. Shown are the first MCA mode by correlating SLP and typhoon track density onto respective time series. The percentage of the explained squared covariance is 72.05% (**a**), 69.92% (**b**) and 65.09% (**c**), respectively. Patterns of other MCA modes in Supplementary Table 1, i.e. including El Niño years, are almost identical to Supplementary Fig. 7 (therefore not shown).

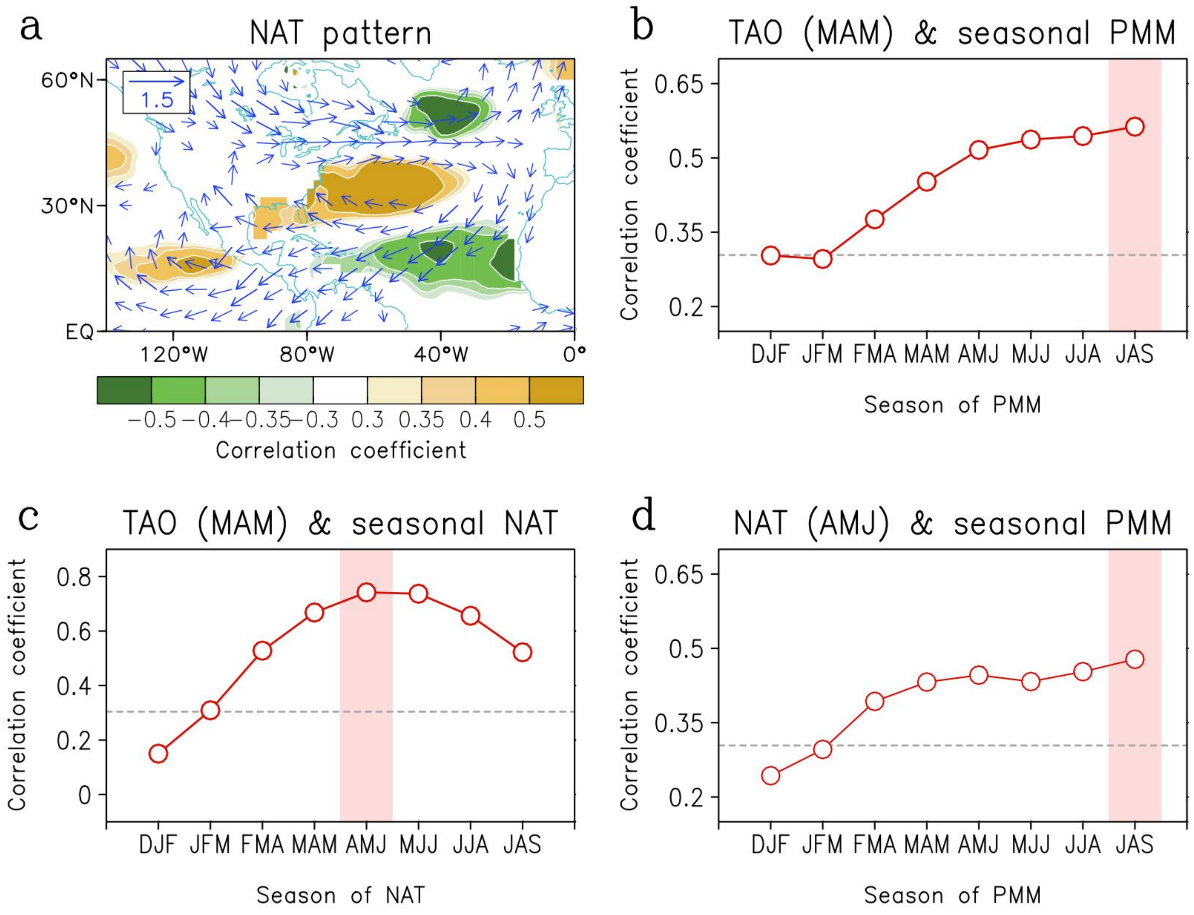

Supplementary Fig. 8. SST evolution related to TAO. a Correlation pattern of SST in April–June (shading) and 850-hPa horizontal wind in March–May (vector) onto the spring TAO index during 1979–2023. **b** Lead-lag correlation of seasonal PMM onto the spring TAO index. The horizontal dashed line denotes the threshold of the 95% confidence level. **c–d** Same as **b** but for seasonal NAT and spring TAO index, and seasonal PMM and April–June NAT index, respectively.

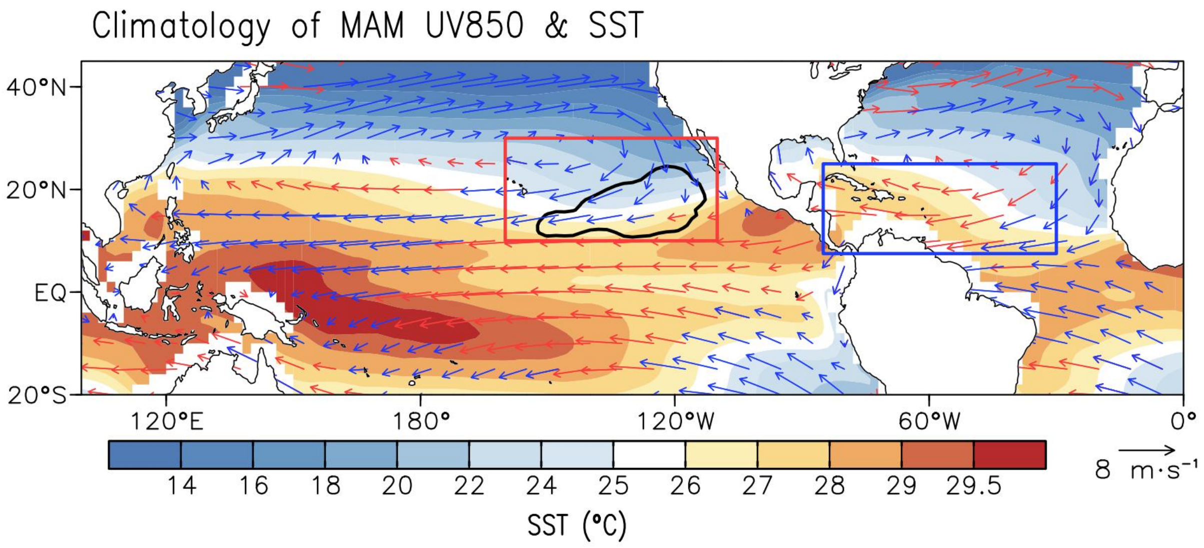

Supplementary Fig. 9. Climatology of SST and 850-hPa horizontal wind over the Pacific and the Atlantic during March–May, 1979–2023. Red (blue) vectors indicate that climatic wind will enhance (weaken) associated with positive TAO (diagnosed by climatic wind speed compared with wind speed plus the regression onto TAO index). Black contour in the Pacific is the 0.5 correlation coefficient of SST onto the July–September PMM index, representing the origin of PMM. Red and blue rectangles show the regions of PMM development and NAT maintenance, respectively.

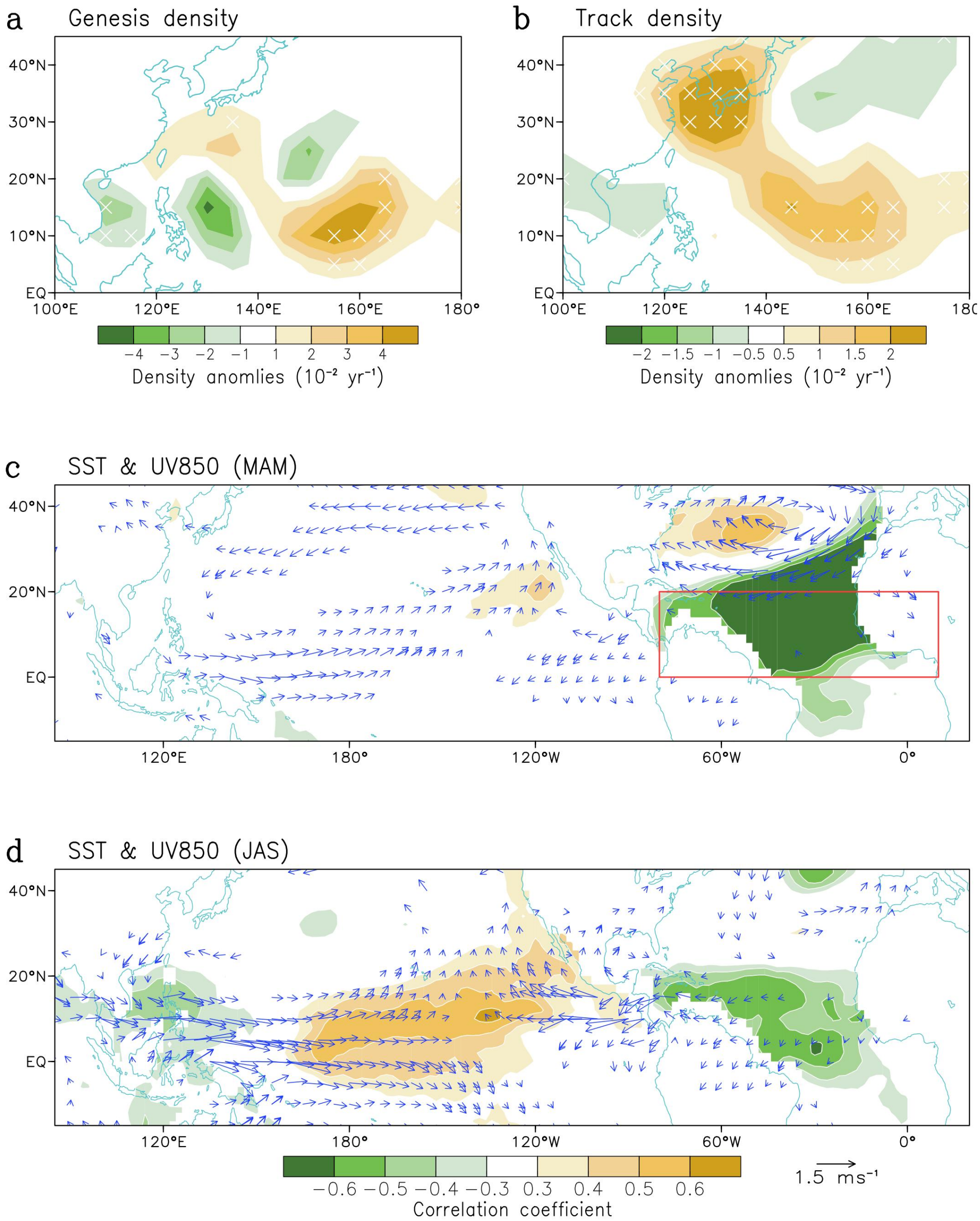

Supplementary Fig. 10. TNA SST and typhoon activity. a Regressions of peak season typhoon genesis density onto the spring Tropical North Atlantic (TNA; mean SST in 80°W–20°E, 0°–20°N, with trend and preceding winter Niño3.4 index removed) index during 1979–2023. **b** Same as **a** but for track density. **c** Correlation of SST (shading) and regression of 850-hPa horizontal wind (vector; $p < 0.05$) during spring (March–April–May) onto the spring TNA index. **d** Same as **c** but SST and horizontal wind are during peak season (July–August–September).

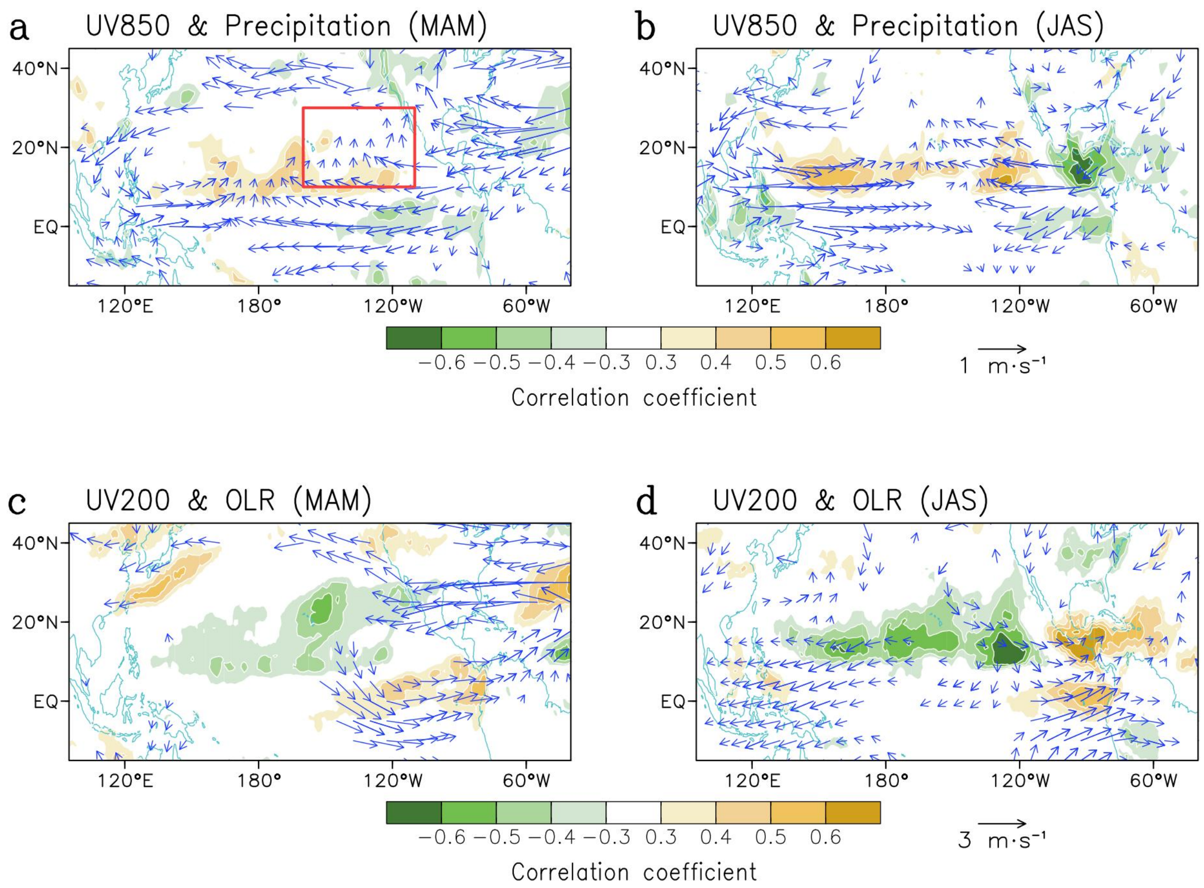

Supplementary Fig. 11. TAO-associated atmospheric anomaly. a Regression of 850-hPa horizontal wind (vector) and correlation of precipitation (shading) during March–May onto TAO index. Red rectangle is the same as in Supplementary Fig. 9. **b** Same as **a** but variables are during July–September. **c**–**d** Same as **a**–**b** but for 200-hPa horizontal wind and outgoing longwave radiation. All the shadings and vectors are significant at the 95% confidence level. Here the data used are shown in Supplementary Methods.

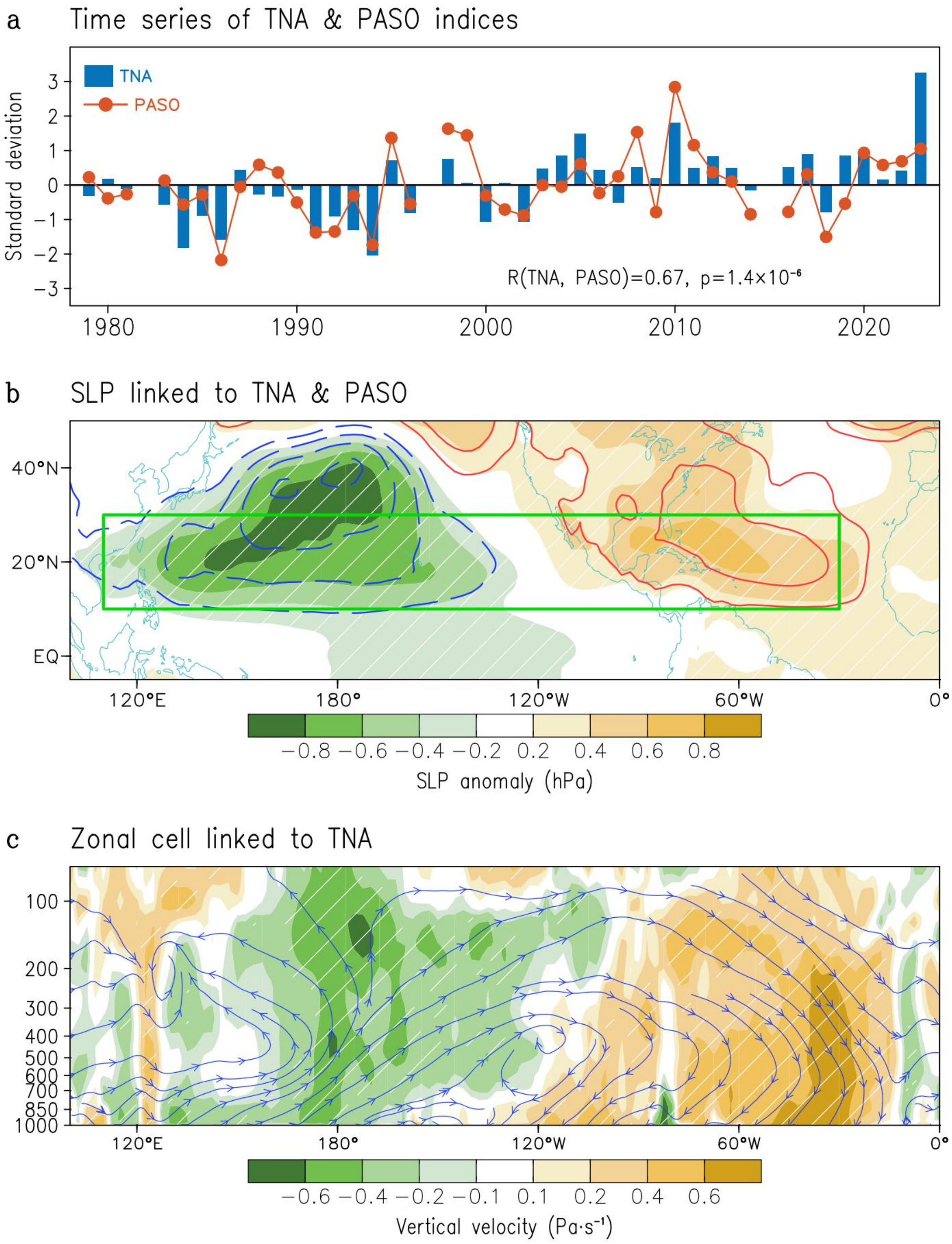

Supplementary Fig. 12. TNA-associated atmospheric anomaly. a Time series of TNA (blue) and PASO (red) during July–September from 1982 to 2023. Correlation between them are marked. Note that after removing the long-term trend, correlation coefficient remains as high as 0.66 ($p < 0.01$). **b** Regression of SLP onto inverse TNA (contour; interval: 0.2 hPa) and reverse PASO (shading). **c** Regression of vertical circulation (streamline) and corresponding vertical velocity (shading) between 10°N–30°N onto inverse TNA index during July–September. Hatching indicates shading passes the 95% confidence level.

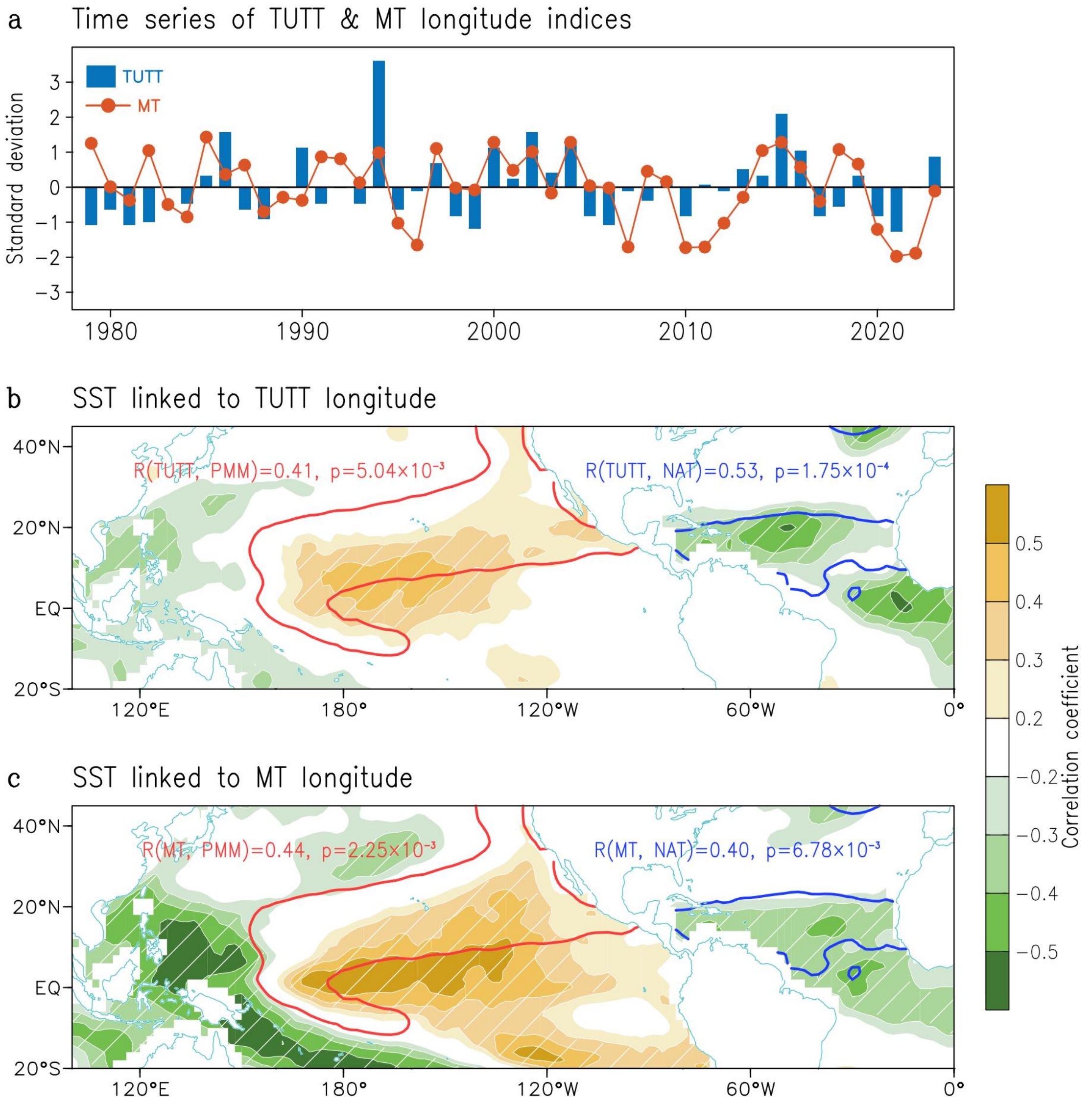

Supplementary Fig. 13. Relationship of zonal shift in TUTT and MT with SST during July–September. a Time series of TUTT (blue) and MT (red) longitude during 1979–2023. **b** Correlation of SST onto TUTT index. Hatching indicates correlation pass the 95% confidence level. Red (blue) contour is the 0.6 (−0.3) correlation of SST onto the July–September PMM index (April–June NAT index). Correlation coefficients between TUTT and PMM/NAT are marked. **c** Same as **b** but for MT index. Here the definitions of TUTT and MT indices are shown in Supplementary Methods.

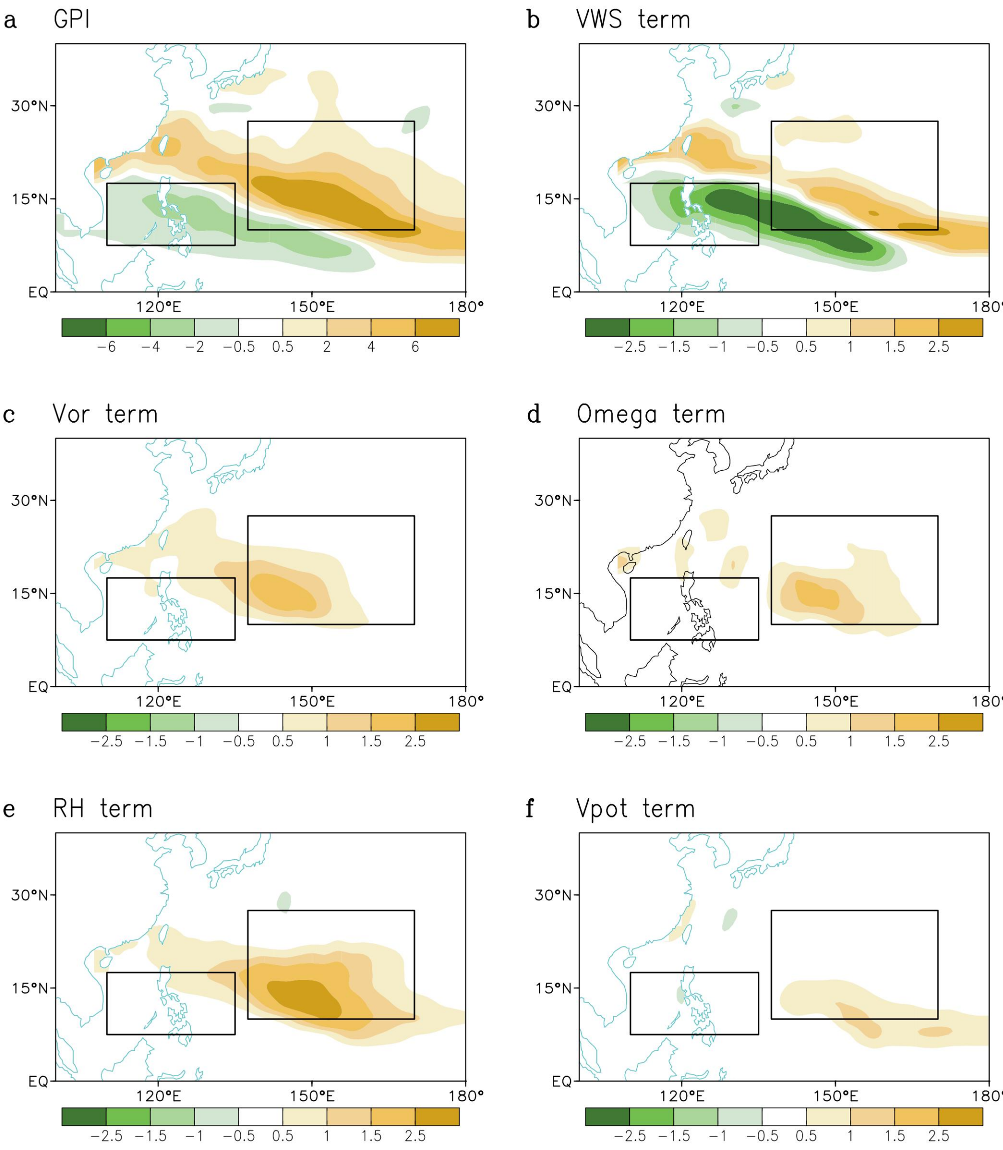

Supplementary Fig. 14. Diagnosis of the GPI associated with the springtime TAO. a–f Regression of peak season GPI **a** and its individual contributing terms during 1979–2023: **b** vertical wind shear (VWS), **c** absolute vorticity (Vor), vertical velocity **d** (Omega), **e** relative humidity (RH), and **f** maximum potential intensity (Vpot) onto the TAO index during spring. Black boxes denote the eastern (137.5°–170°E, 10°–27.5°N) and western (110°–135°E, 7.5°–17.5°N) analysis regions.

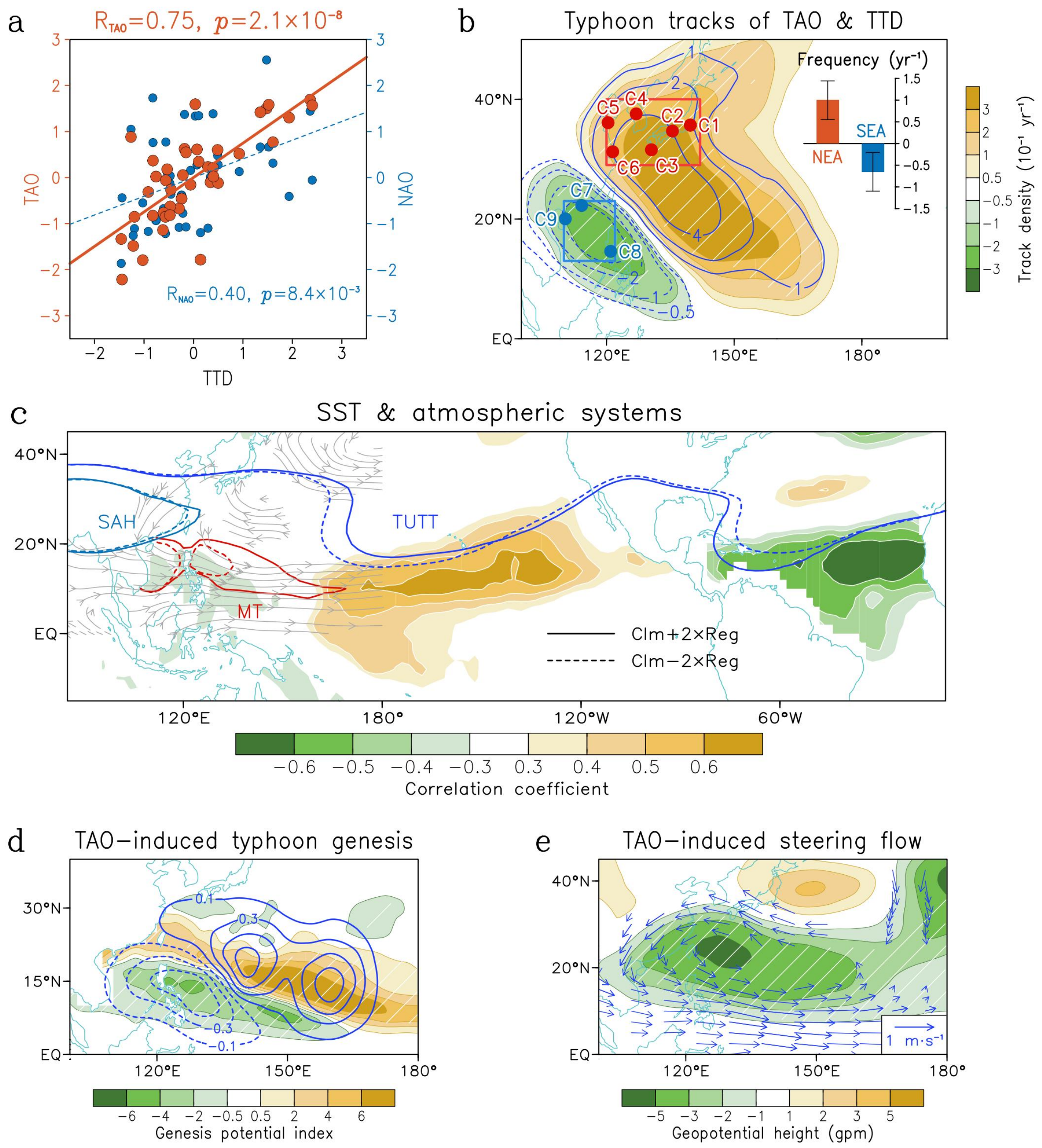

Supplementary Fig. 15. TAO modulations on typhoon and circulation. Same as Fig. 2a–b, 3a–c, but trend is removed. a Scatter diagram of the spring TAO (red) and extratropical NAO (blue) against the TTD of peak season typhoon track density. Correlation coefficient (R) between the TAO (extratropical NAO) and TTD is marked. Straight lines are linear least-square fits. **b** Regressions of peak season typhoon track density onto the TAO (shading) and TTD (contours) indices. **c** Shading in the Pacific (Atlantic) is the correlation pattern of SST in July–September onto the simultaneous PMM index (April–June NAT index). Streamlines are the regressed 850-hPa horizontal wind onto the PMM+NAT index (WNP region). Three atmospheric systems are shown: monsoon trough (MT; represented by the relative vorticity of 3×10^{-6} s^{-1} at 850 hPa), tropical upper-troposphere trough (TUTT; represented by the 12420-gpm geopotential height at 200 hPa) and South Asian high (SAH; represented by the 12500-gpm geopotential height at 200 hPa). Solid (dashed) contours are

calculated as the climatology plus (minus) the double regressions onto the PMM+NAT index. **d** Regressions of peak season Genesis Potential Index (shading) and typhoon genesis density (contour; 10^{-1} yr^{-1}) on the spring TAO index. **e** Regressions of peak season 700-hPa geopotential height (shading) and steering flow (vector; $p < 0.05$) onto the spring TAO index. Hatching indicates that it passes the 95% confidence level.

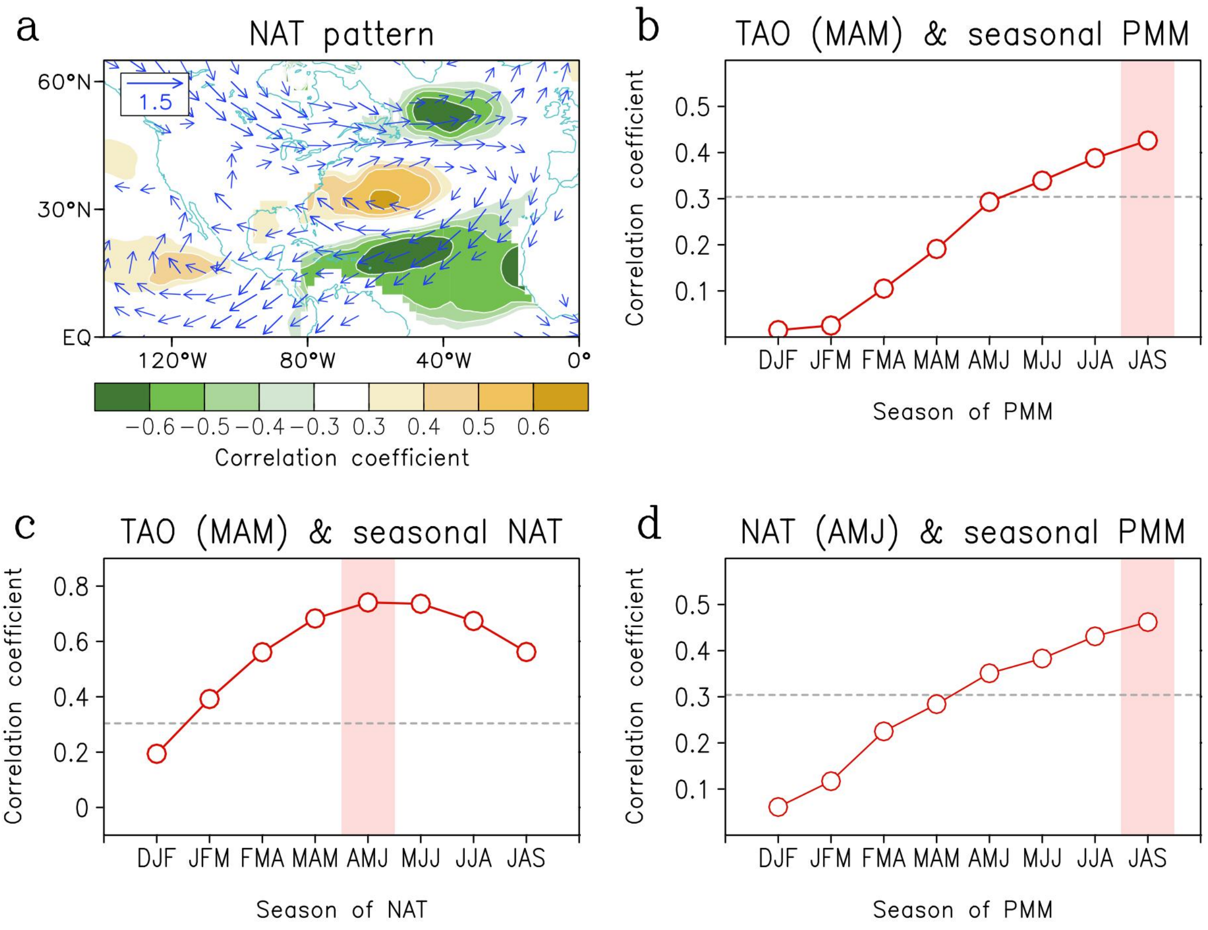

Supplementary Fig. 16. SST evolution related to TAO. Same as Supplementary Fig. 8, but trend is removed. a Correlation pattern of SST in April–June (shading) and 850-hPa horizontal wind in March–May (vector) onto the spring TAO index. **b** Lead-lag correlation of seasonal PMM onto the spring TAO index. The horizontal dashed line denotes the threshold of the 95% confidence level. **c–d** Same as **b** but for seasonal NAT and spring TAO index, and seasonal PMM and April–June NAT index, respectively.

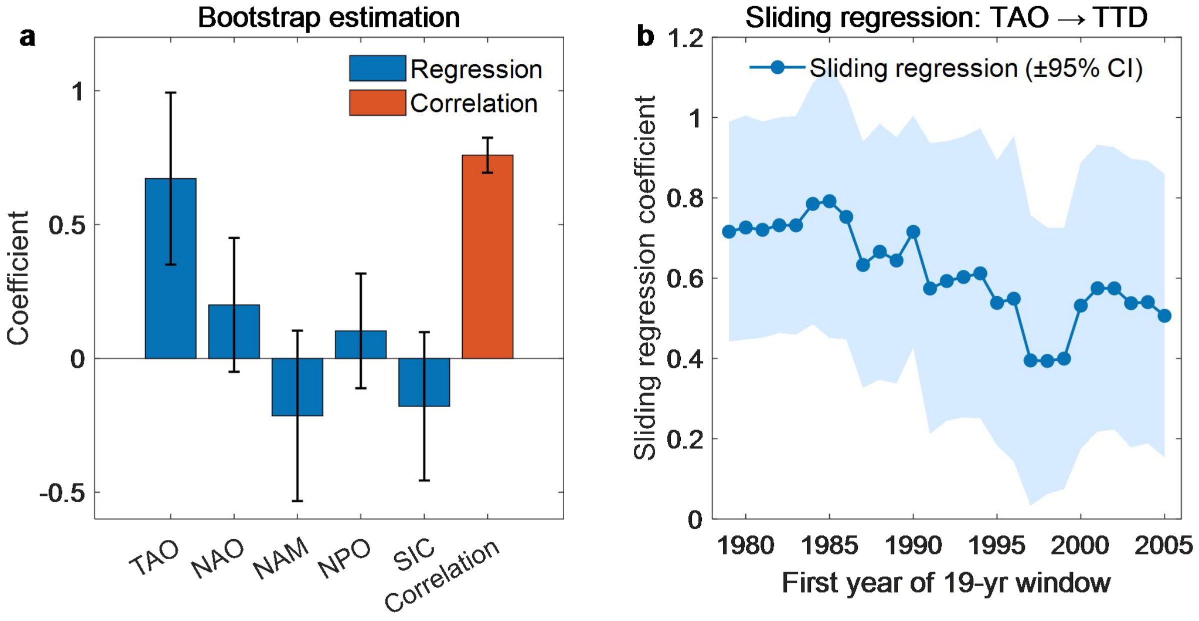

Supplementary Fig. 17. Stability of TAO-TTD relationship. a Bootstrap estimation (see Supplementary Methods) of regression coefficients and correlation coefficient for predicting TTD using five climate indices (TAO, NAO, NAM, NPO, SIC) and based on data during all years from 1979 to 2023. Blue bars indicate the mean regression coefficients (including intercept), with black error bars representing the 95% confidence interval. The red bar shows the mean correlation between observed and predicted TTD with its 95% confidence interval. **b** Sliding regression between TAO and TTD over a 19-year moving window. The blue line with markers shows the regression in each window, and the shading indicates the 95% confidence interval.

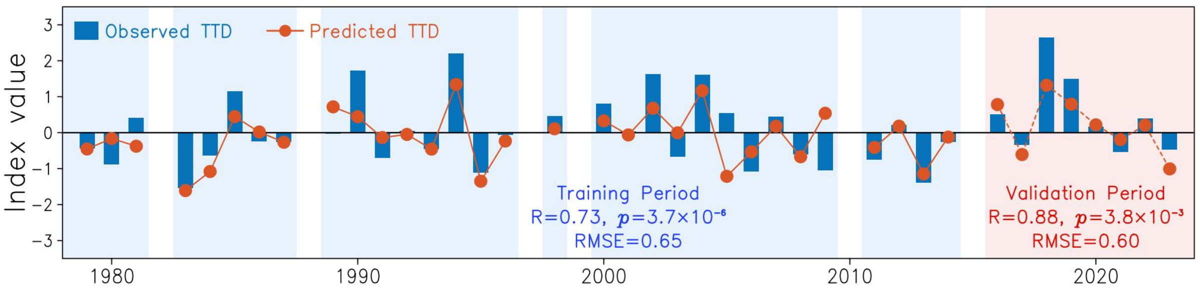

Supplementary Fig. 18. Same as Fig. 4a, but further excluding three extreme La Niña years (1988, 1999 and 2010).

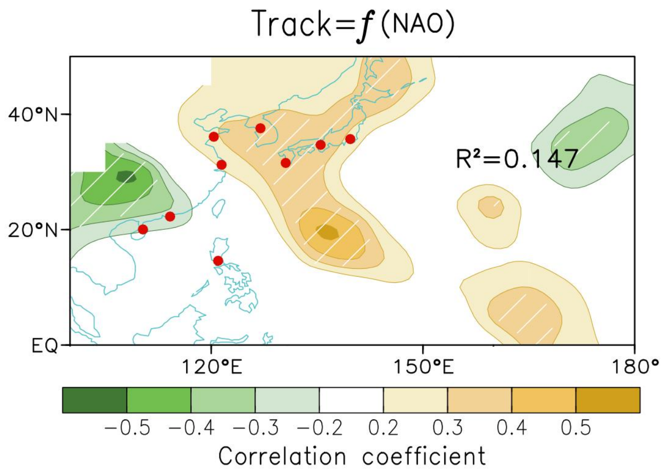

Supplementary Fig. 19. Same as Fig. 4b, but for NAO.

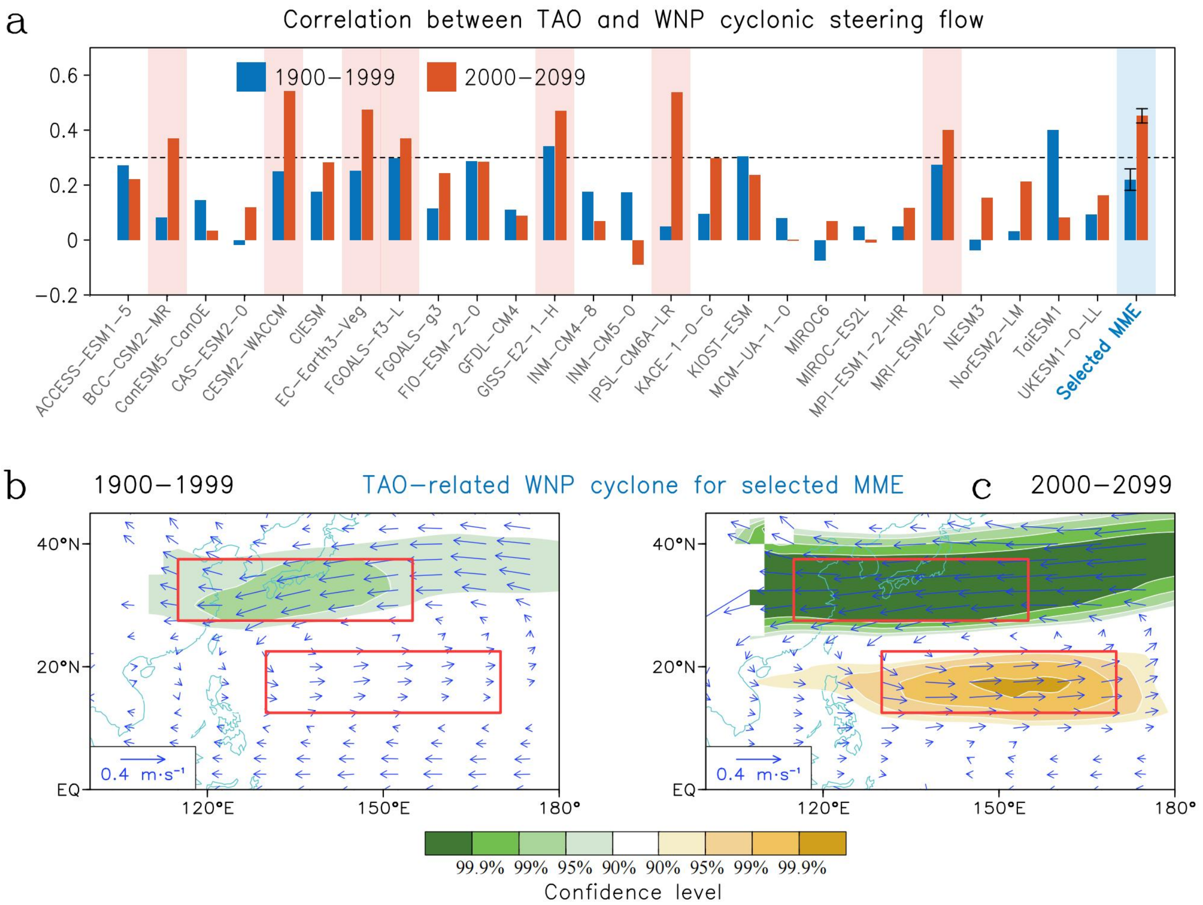

Supplementary Fig. 20. Relationship between TAO and WNP cyclone in CMIP6 models. **a** Correlation coefficient between spring TAO and peak season cyclonic steering flow in the WNP during 1900–1999 (blue bars) and 2000–2099 (red bars). The selected 7 ensembles are in the shaded background with error bar showing the 95% confidence interval for the selected MME. The steering flow index is defined as zonal steering flow difference between (12.5°N–22.5°N, 130°E–170°E) and (27.5°N–37.5°N, 115°E–155°E). **b** Mean of regression patterns of peak season steering flow (vector) onto the spring TAO index during 1900–1999 for the selected 7 ensembles. Shading is the correlation of zonal component of steering flow onto the TAO index. **c** Same as **b** but during 2000–2099.

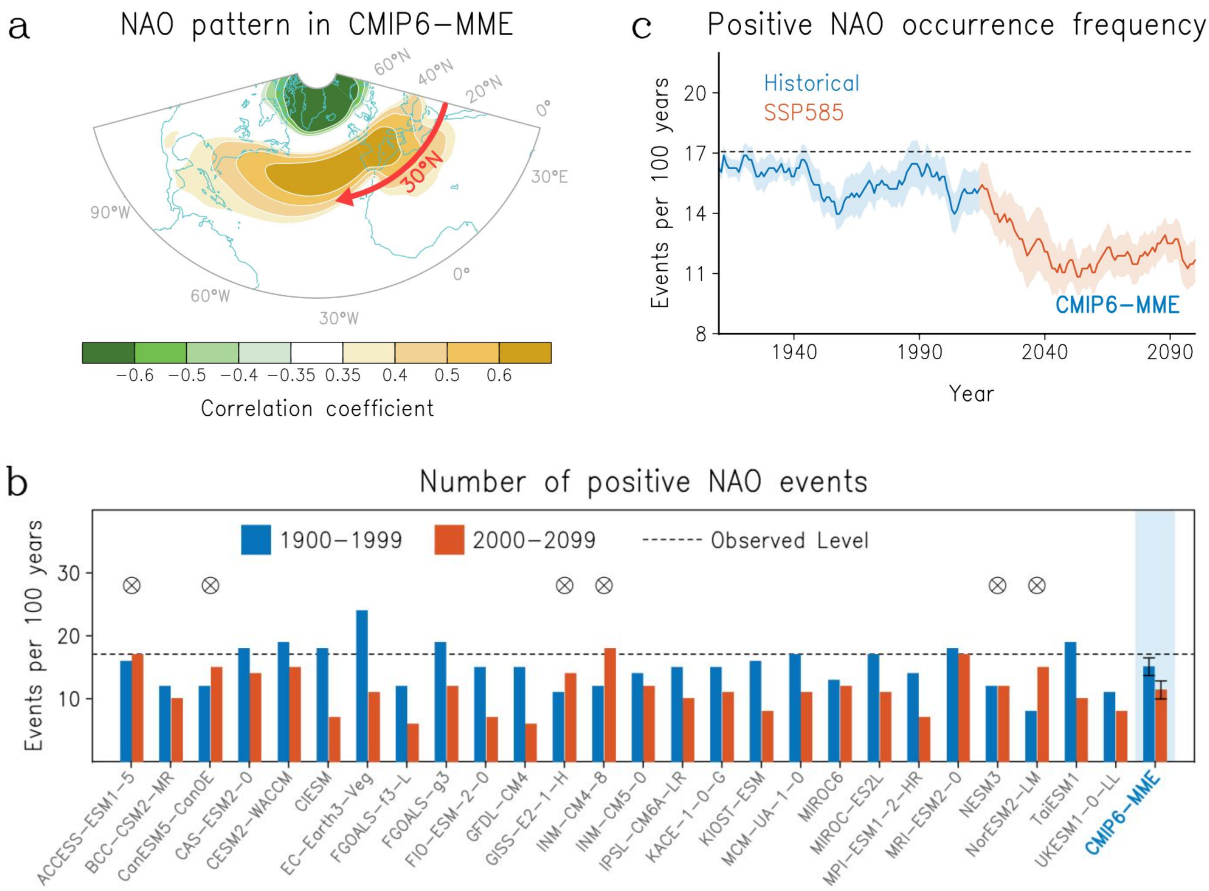

Supplementary Fig. 21. NAO in CMIP6 multi-member ensemble. Same as Fig. 5 but for NAO. Cross-in-circle marks the models that do not simulate a decrease in positive NAO events.

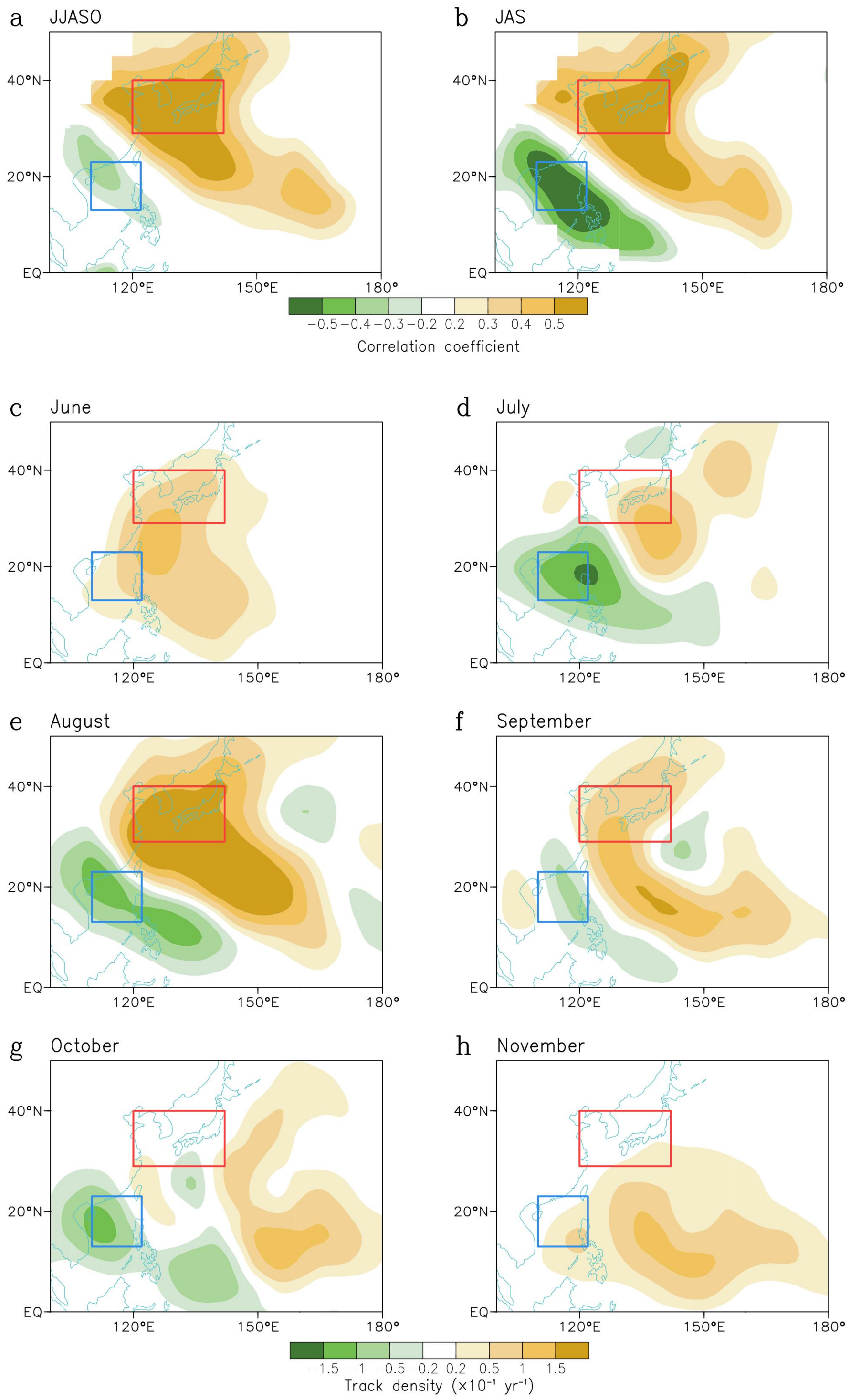

Supplementary Fig. 22. Seasonal and monthly typhoon anomaly in response to TAO. Correlation of typhoon track density in (**a**) June–October (JJASO) and (**b**) July–September (JAS), 1979–2023. Regression of typhoon track density in (**c**) June, (**d**) July, (**e**) August, (**f**) September, (**g**) October and (**h**) November onto the spring TAO index. Rectangles are the same as in Fig. 2b.

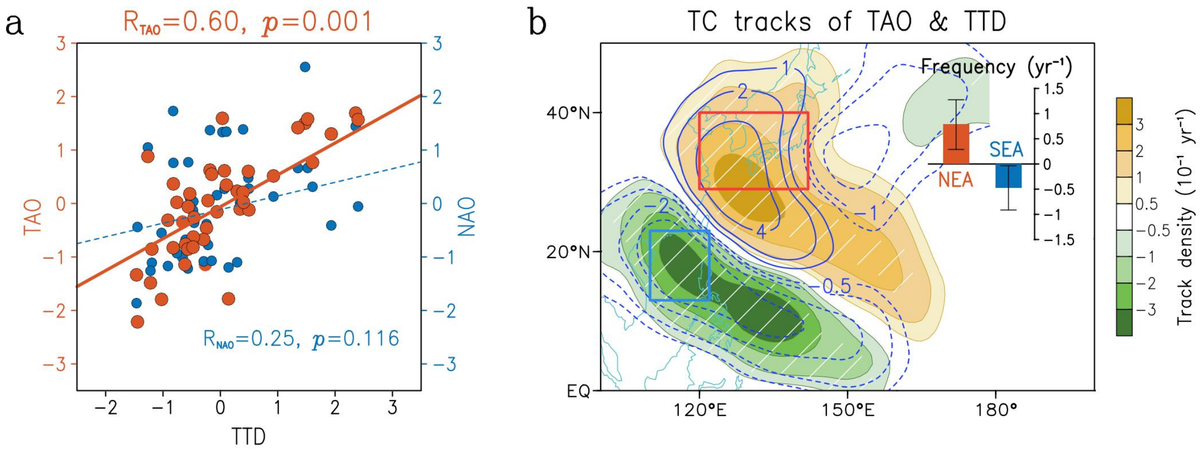

Supplementary Fig. 23. Relationship between TAO and all TC activity during peak season. Same as Fig.2a–b but for TCs with V_{max} > 17.2 m s^{-1}.

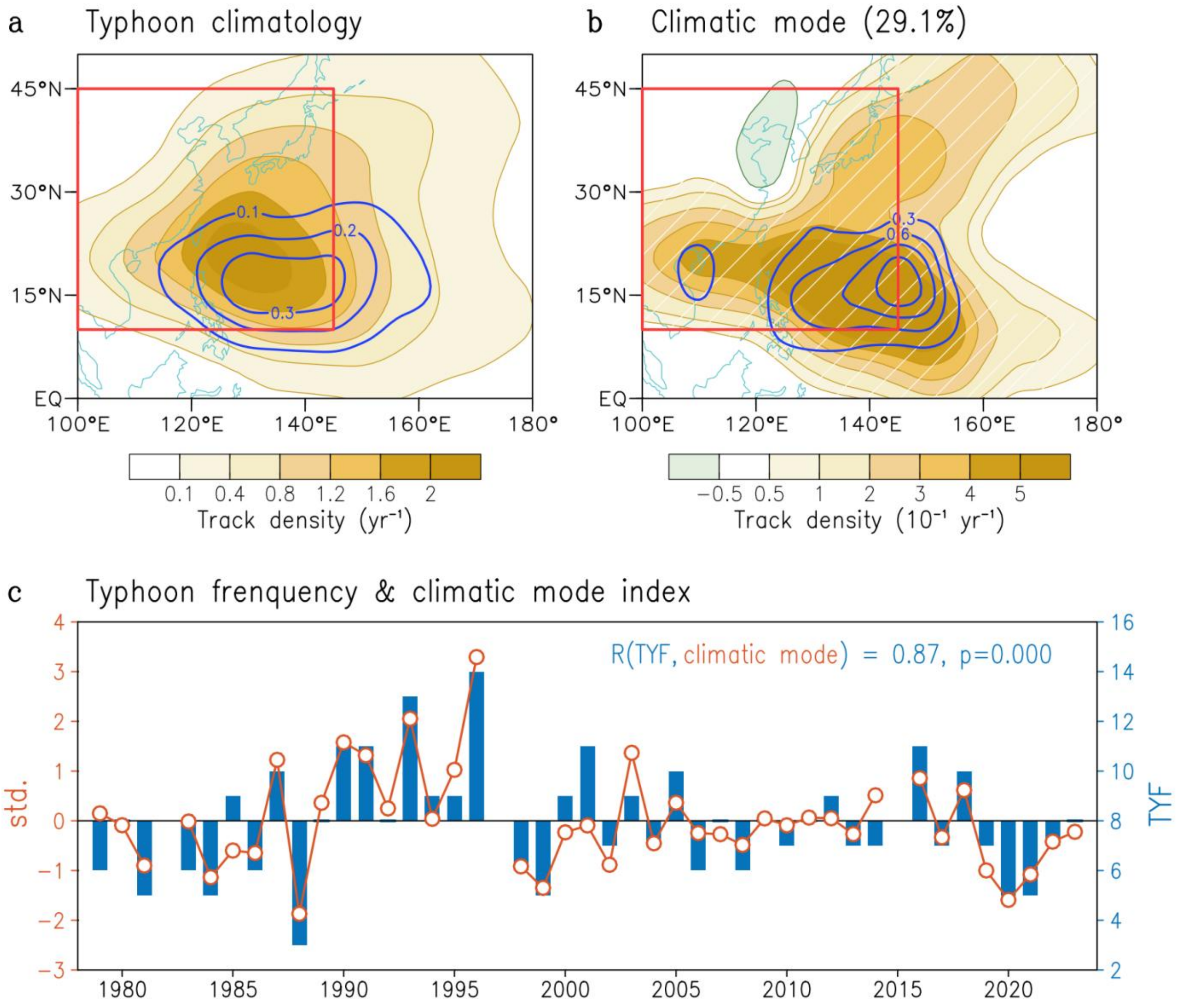

Supplementary Fig. 24. The original first EOF mode of East Asian typhoon. a Climatology of typhoon genesis (contour; yr^{-1}) and track density (shading) during July–September, 1979–2023. **b** Regression of typhoon genesis (contour; 10^{-1} yr^{-1}) and track density (shading) onto the climatic mode (leading EOF mode using the raw data) index. Box indicates the region used for EOF analysis. **c** Time series of climatic mode (red) and typhoon frequency inside the box (TYF; blue).

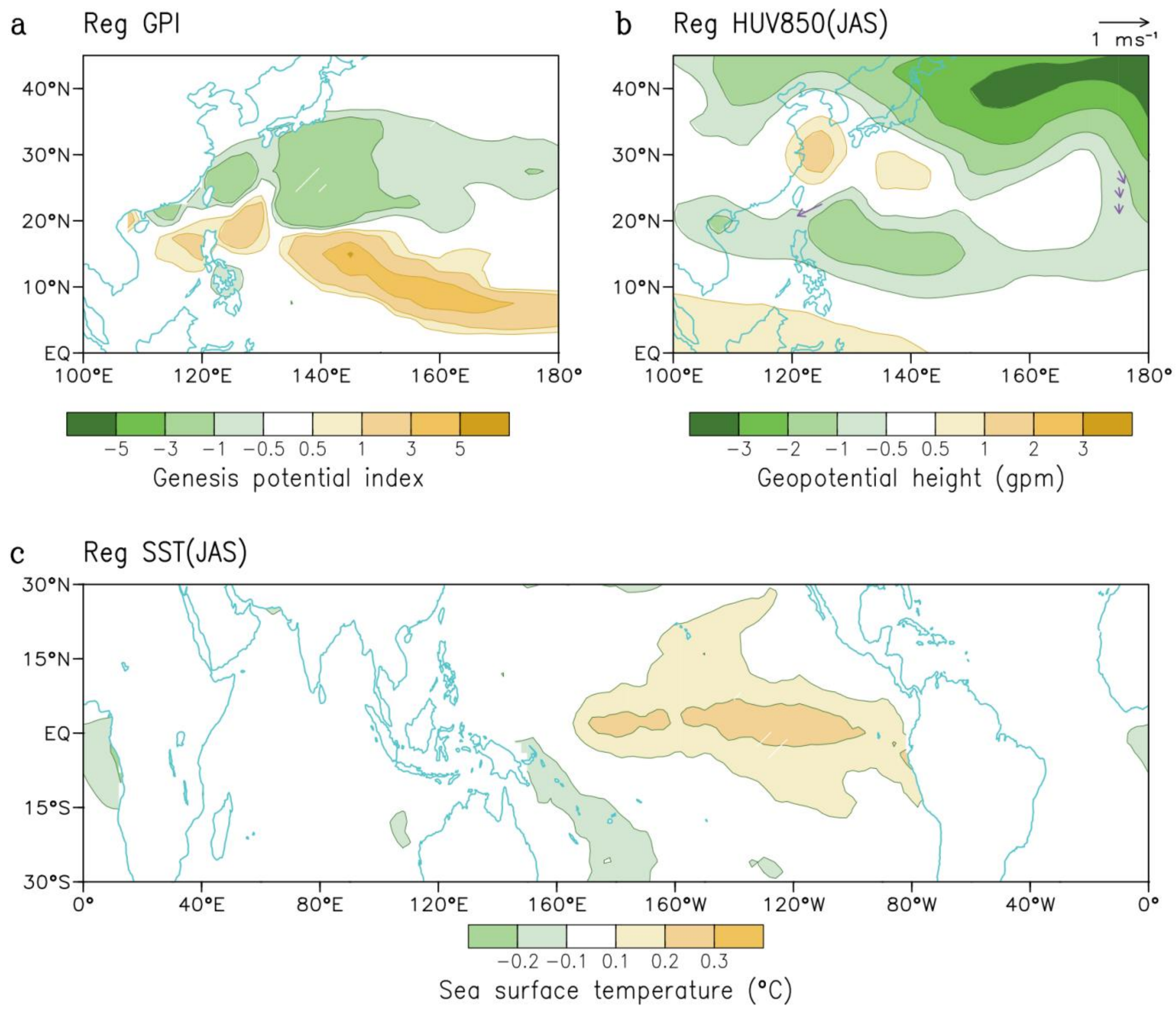

Supplementary Fig. 25. Anomalies associated with the original first EOF mode of East Asian typhoon. Regression of genesis potential index (**a**), geopotential height (shading) and horizontal wind (vector; $p < 0.01$) at 850 hPa (**b**), and SST (**c**) during July–September 1979–2023 onto the climatic mode index. Hatching indicates that regression passes the 99% confidence level.

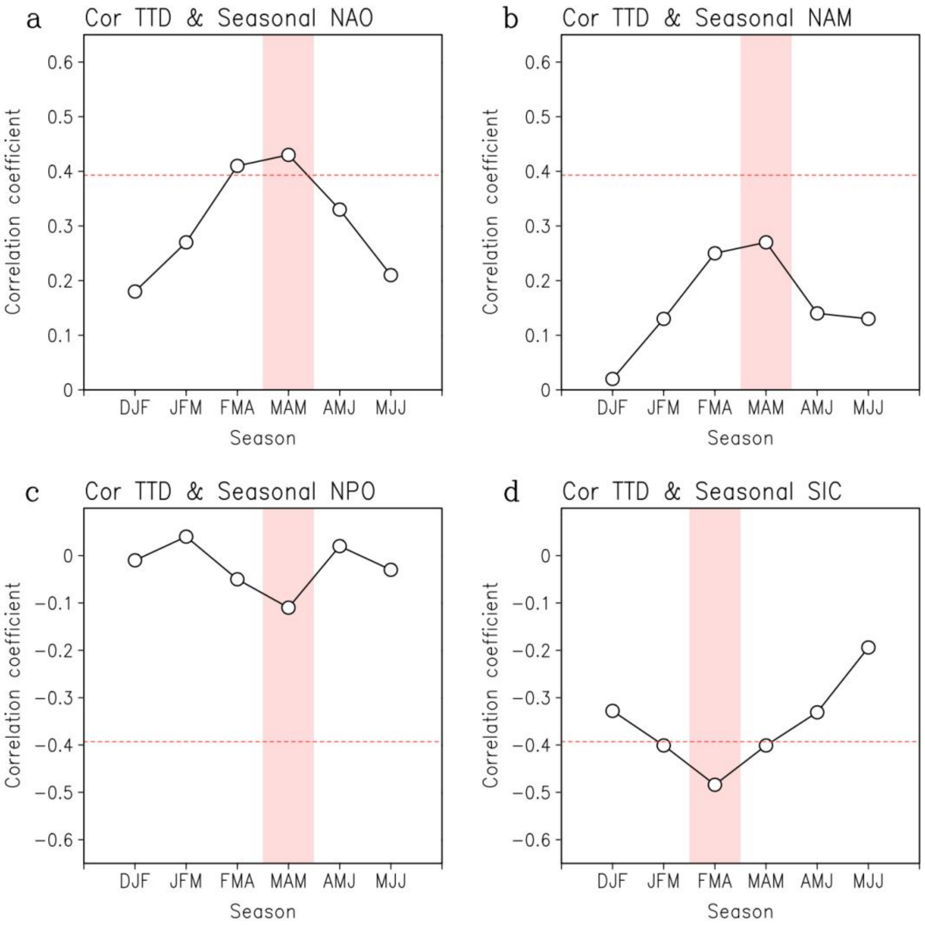

Supplementary Fig. 26. Seasonal relationship between predictors and TTD. Correlation coefficients of peak season TTD index with seasonal NAO (**a**), NAM (**b**), NPO (**c**) and SIC (**d**) indices during 1979–2023. The horizontal dashed line is the threshold of the 99% confidence level. Shading denotes the season of the predictors we used in the prediction model.

Supplementary References:

1. Adler, R. F. et al. The Global Precipitation Climatology Project (GPCP) monthly analysis (new version 2.3) and a review of 2017 global precipitation. Atmosphere 9, 138 (2018).
2. Lee, H. T. Climate algorithm theoretical basis document (C-ATBD): Outgoing longwave radiation (OLR)-daily. NOAA's Climate Data Record (CDR) Program. CDR-ATBD-0526 111, 2804-2822 (2014).
3. Wu, L., Wang, C. & Wang, B. Westward shift of western North Pacific tropical cyclogenesis. Geophys. Res. Lett. 42, 1537-1542 (2015).
4. Zhang, C. et al. Perspective on landfalling frequency and genesis location variations of southern China typhoon during peak summer. Geophys. Res. Lett. 46, 6830–6838 (2019).
5. Austin, P. C. & Tu, J. V. Bootstrap methods for developing predictive models. *The American Statistician* **58**, 131-137 (2004).
